# MicroRNA expression profiling in exosomes derived from gastric cancer stem-like cells

**DOI:** 10.18632/oncotarget.21288

**Published:** 2017-09-27

**Authors:** Zhan-Peng Sun, An-Qi Li, Wen-Huan Jia, Sen Ye, Grace Van Eps, Jian-Min Yu, Wei-Jun Yang

**Affiliations:** ^1^ College of Life Sciences, Zhejiang University, Hangzhou, China; ^2^ Key Laboratory of Conservation Biology for Endangered Wildlife of The Ministry of Education, Zhejiang University, Hangzhou, China

**Keywords:** gastric cancer, cancer stem cells, exosomes, microRNAs, high-throughput sequencing

## Abstract

Cancer stem-like cells (CSCs) have been identified as the initial cell in formation of cancer. Quiescent CSCs can “hide out” from traditional cancer therapy which may produce an initial response but are often unsuccessful in curing patients. Thus, levels of CSC in patients may be used as an indicator to measure the chance of recurrence of cancer after therapy. The goals of our work are to develop specific exosomal miRNA clusters for gastric CSCs that can potentially predict which patients are at high risk for developing gastric cancer (GC) in order to diagnose GC at an early stage. Here, upon sorting gastric CSCs, we initially isolated and characterized exosomes secreted by both gastric CSCs and their differentiated cells (DCs). By deep sequencing of each exosomal miRNA library, 11 typical differentially expressed miRNAs were identified as signature miRNAs for CSC. Gene target prediction, GO annotation and KEGG pathway enrichment analysis showed possible functions associated with these signature miRNAs. Hence, upon research of exosomal miRNAs that would influence behavior of tumor cells and their microenvironment, this study shows that a specific miRNA signature is present in CSCs, and implies that a potential miRNA biomarker reflecting the stage of gastric cancer progression and metastasis could be developed in the foreseeable future.

## INTRODUCTION

Gastric cancer (GC) is the fourth most common cancer and the second leading cause of cancer mortality in the world [1]. Although gastric cancer has a good prognosis when detected at an early stage [2], the survival rate dramatically decreases in patients with advanced or aggressive tumors because of difficulty in early diagnosis and recurrences after surgical resection that lead to the development of locoregional recurrence and/or distant metastasis [3, 4]. The aggressiveness of advanced tumors is regarded as the reason for activation of hypothetical existing gastric CSCs, which is defined as cells within a tumor that possess the capacity for self-renewal and differentiation, as well as innate resistance to chemotherapy and radiation [5, 6]. With increasing discovery of CSCs in many different gastric cancer cell lines, the hypothesis that cancer stem cells are involved in gastric cancer has been proposed [7], and led to a series of experiments on the purification and characterization of CSCs from gastric cancer cells [8-12]. Previously published studies have defined CD44+ subpopulations as gastric cancer stem-like cell pools in poorly differentiated cell lines, which confirms the hierarchical organization of immortalized cell lines [12]. Targeting of CSCs facilitates comprehension of the origin of tumors and may further lead to novel therapies with better clinical outcomes (low disease recurrence after definitive therapy) [13].

Exosomes are biological nanovesicles (30–150 nm in diameter), containing a wide range of functional proteins, mRNAs and microRNAs (miRNAs) [14-17]. Advanced carcinoma is reported to produce a large quantity of exosomes in contrast to early stage tumor or normal tissue cells [18, 19]. Tumor-derived exosomes are released locally and into circulation to interact with a variety of target cells [20, 21]. These exosomes promote tumor progression through communication between the tumor and surrounding stromal tissue [22], as well as activation of proliferative and angiogenic pathways [23], by bestowing immune suppression [24, 25] and initiation of pre-metastatic sites [26]. Since exosomes contain cell-type specific proteins and genetic material from their parental cells, exosomes, as well as the materials they contain, are being explored as a prognostic indicator of advancing malignancy in several types of cancer [27]. The concentrations of exosomes correlate with increased malignant behavior of the cancer [28-30]. Therefore, cancer-specific proteins and microRNA signatures in exosomes were found to serve as biomarkers for different tumor types and stages [31-33]. Previously published studies have identified several exosomal miRNA clusters as cancer prognostic markers and/or grading basis, and these miRNAs also promotes in cancer progression [34-37].

Several studies have identified distinct exosomal miRNA signatures in gastric cancer [38-40], but identification and analysis of exosomes and miRNAs in gastric CSCs has not been published yet. Moreover, none so far have performed a comparative differential profiling of exosomal miRNAs between CSCs and their progeny. As the increasing appearance of cancer stem cells, it can be considered as a sign of GC progression and a bad signal of cancer recurrence. The lack of studies on tracking CSCs makes the diagnosis of progression and stages of GC a difficult problem.

Herein, we performed high throughput sequencing of miRNAs in exosomes derived from gastric cancer stem-like cells and their differentiated counterparts. The miRNAs in exosomes were analyzed with the identification of signature miRNAs. These data may shed light on the relationship between stem cells and gastric cancer on the molecular level, further enhance our understanding of gastric cancer progression, and help develop potential biomarkers that may be useful for both diagnosis and prognosis of gastric cancer progression.

## RESULTS

### Isolation and identification of gastric cancer stem-like cells (CSCs)

**Figure 1:**
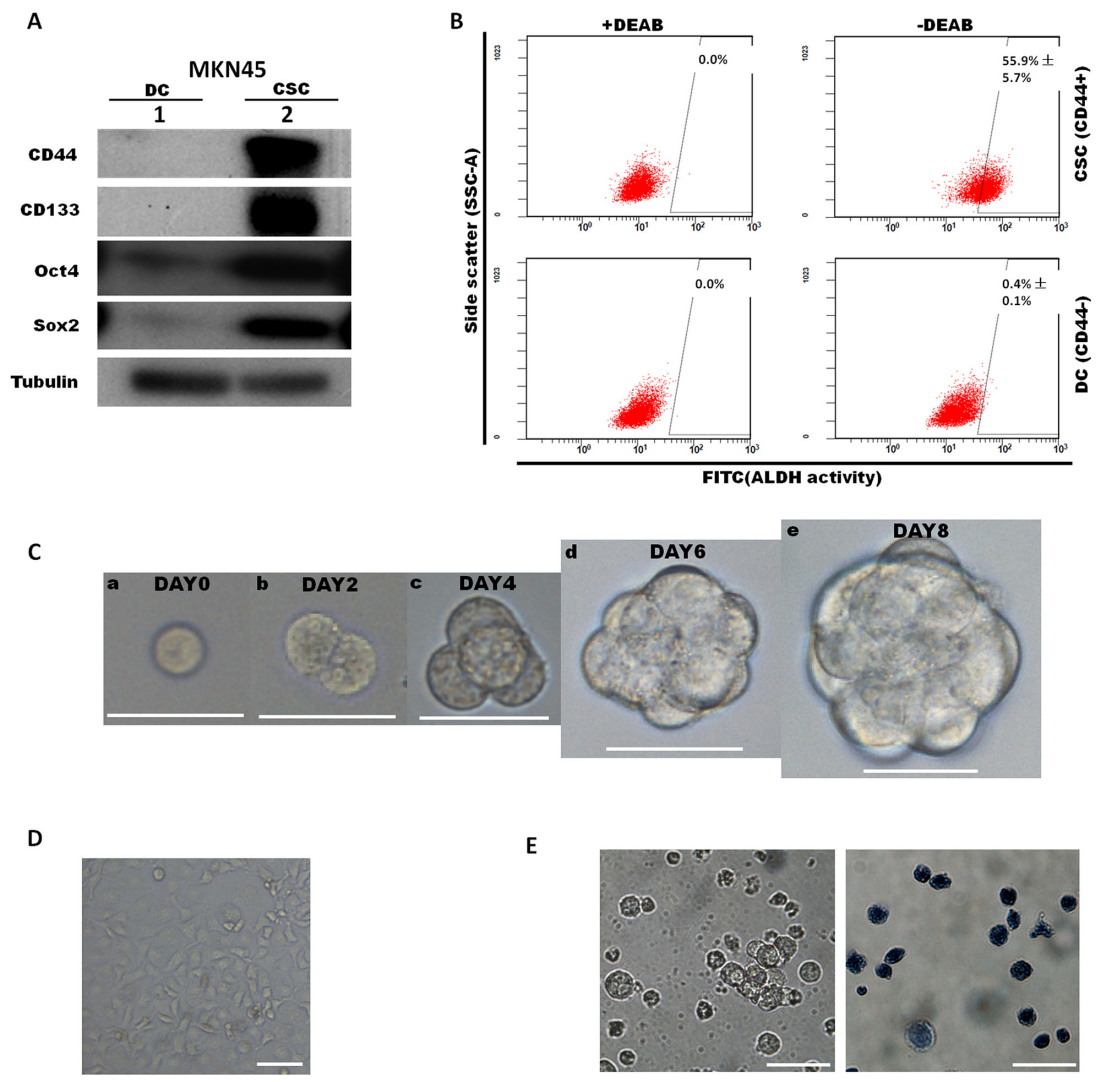
Characterization of cancer stem-like cells sorted from the MKN45 cell line according to CD44 expression By use of cell surface marker CD44, cancer stem-like cells (CSCs) population and differentiated cells (DCs) population were isolated and FACS-sorted from MKN45 cell line. **(A)** Expression levels of CD44, CD133, Sox2, Oct4 were determined by western blot between sorted MKN45 CSC population and DC population. **(B)** Cells dissociated from gastrospheres (passage1, on day 8) were stained with ALDEFLUOR reagent and analyzed by flow cytometry. Diethylaminobenzaldehyde (DEAB) was used to inhibit ALDH activity, to show the specificity of detection. Representative images of flow cytometry analyses and quantification were shown. Values indicate the mean ±s.d. of positive cells (n=3). **(C)** Formation of gastrospheres were observed from day0 to day 8 (a-e) by plating CD44+ cells at clonal density in low-attachment surface culture, Scale bars: 50 μm. **(D)** CD44- cells represented for differentiated cells (DCs), were isolated by FACS and plated on adherent surface. Scale bars: 50μm. **(E)** CD44- cells became apoptotic (trypan blue positive) after 2 weeks under CSC culture condition. Left, bright field; right, trypan blue staining.

Based on several previously published studies, cancer stem-like cells (CSCs) and their differentiated progeny cells (DCs) both existed in three human gastric cancer cell lines (MKN-45, MKN-74, and NCI-N87). Several markers were used to isolate CSCs, such as CD24, CD44 and CD133. In the present study, flow cytometry and FACS were utilized to separate gastric CSCs and DCs defined by cell surface marker CD44 in the MKN45 cell line. We fractionated MKN45 by FACS sorting for 1% CD44 strongly positive (CD44+) population as CSCs and CD44 negative (CD44-) population as DCs. Next, subpopulations of CD44+ and CD44- cells were collected. Then we performed western blot by using antibody against stemness markers CD44, CD133, Oct4 and Sox2 to validate the effectiveness of FACS fractionation (Figure 1A). Results revealed that CD44 was exclusively enriched in CSCs, while other markers were also highly expressed in the CD44+ population, which demonstrated that CD44+ CSCs were reliably isolated and showed stem-like molecular feature. The spheroid colony formation that involves culturing candidate CSCs under non-adherent conditions in serum-free medium is a typical approach to indicate the self-renewal ability which is an important phenotype of CSCs *in vitro*. After *in vitro* culture for 6–8 days in CSC medium, approximately 95% of FACS-sorted CD44+ cells produced spheroid colonies (Figure 1C), the stemness of gastrospheres (passage1) were further characterized by ALDH activities under staining with ALDEFLUOR reagent and analyzing by flow cytometry. Approximately ∼55.9% CSC cells showed ALDH activities, while only approximately 0.4% of DCs were detectable for ALDH (Figure 1B). Gastrospheres could be enzymatically dissociated to single cells, which in turn gave rise to secondary spheres for more than 20 passages. The results of trypan blue staining showed that the CD44+ spheroid colony-forming cells remained alive after 5 weeks, while most of the CD44- cells, that underwent terminal differentiation, were trypan blue positive after two weeks under non-adherent serum-free culture (Figure 1E). To validate our *in vitro* results, we further performed transplantation of FACS-sorted CSC and DC cells into SCID mice. We found that CD44+ CSC (500-5000 injected cells) generate tumors after 8-12 weeks, while the CD44- DC did not generate any tumors on the other side (Supplementary Figure 4). Above results suggested that FACS-sorted CD44+ spheroid colony-forming cells are consistent with a CSC phenotype.

### Isolation and characterization of CSC-exosomes and DC-exosomes

**Figure 2:**
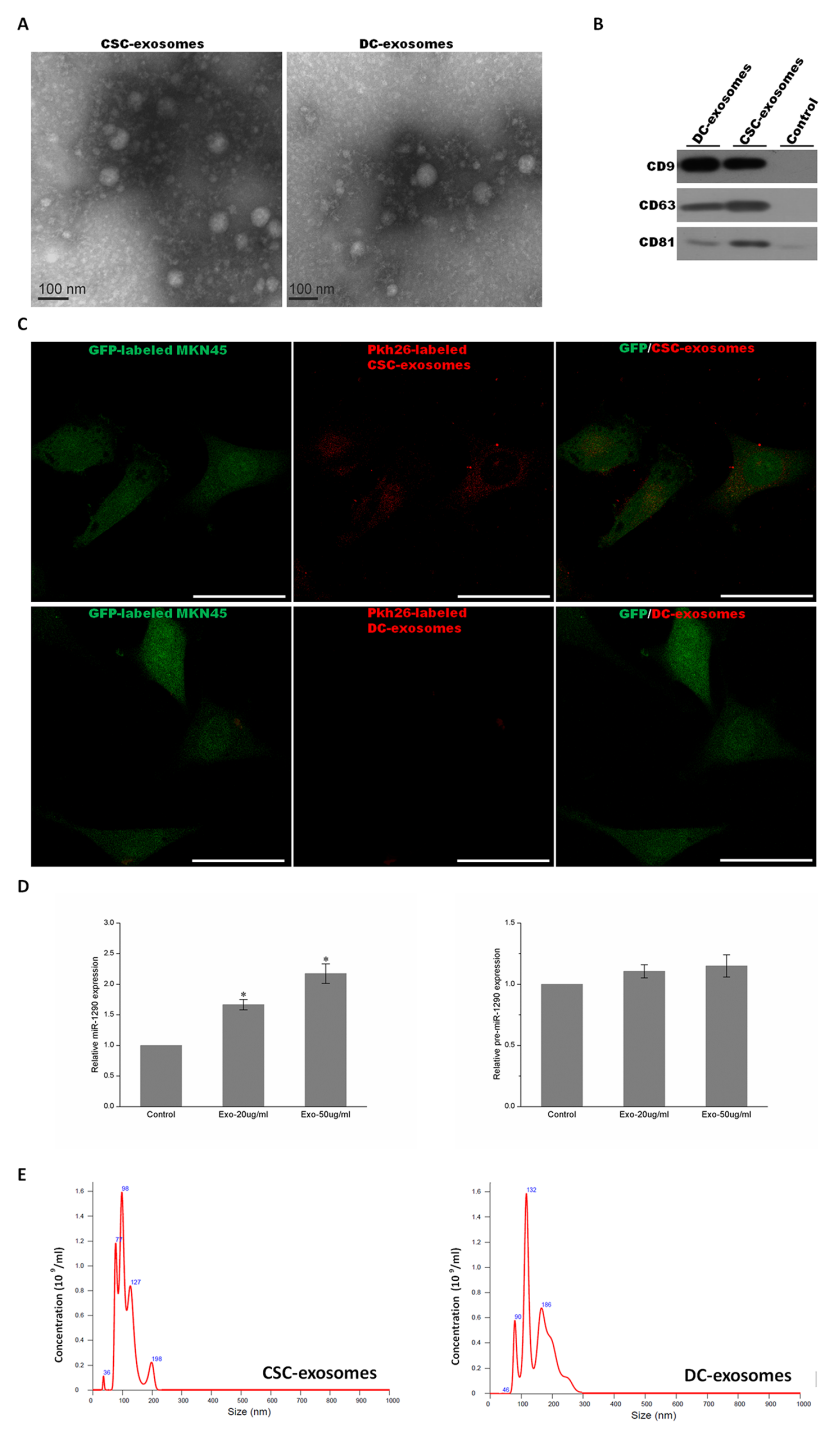
Characterization of exosomes **(A)** Electron micrograph of CSC and DC exosomes. The image shows small vesicles of approximately 50-80nm in diameter. The scale bar indicates 100nm. **(B)** Western blot characterized exosomes derived from CSCs and DCs using antibodies against exosomal protein markers (CD9, CD63 and CD81). Control: concentrated medium supernatant from MKN45. **(C)** Exosomes were initially labeled with Pkh-26. Immunofluorescent analysis of CSC-exosomes treated and DC-exosomes treated MKN45 cells, showing CSC-exosomes but not DC-exosomes can incorporate and label the original MKN45 cells. Scale bar: 50 μm. **(D)** MiR-1290 expression levels in MKN45 treated with 0, 20 and 50 μg/ml of CSC-exosomes for 24 hours were determined by quantitative real-time PCR analysis (left plot). Pre-miR-1290 expression in MKN45 treated with different amounts of CSC-exosomes (right plot). Values are the mean ± SD of 3 independent experiments ^*^p ≤ 0.05; ^**^p ≤ 0.01. **(E)** The diagram shows the size and concentration of exosomes derived from CSCs (left) and DCs (right) by use of nanoparticle tracking analysis (NTA).

In addition to body fluids such as serum and plasma from peripheral blood, exosomes are also found in the medium of cultured cells [41, 42]. In this study, for the purpose of profiling exosomal miRNAs, after CSC and DC cells were grown for 5 days and 48 hours (Figure 1C and 1D), serum-free medium of CD44+ gastrosphere (in CSC culture) and CD44- differentiated progeny (30% exosome-depleted FBS with RPMI1640 medium) was collected, purified by successive centrifugation, then CSC-exosomes and DC-exosomes were isolated by use of ExoquickTM kit respectively, and their purity was confirmed under a transmission electron microscope and using western blot. The results of transmission electron microscope investigation showed two types of exosomes were small (50–150nm diameter) spherical vesicles, and consistent with the known morphology approximately within the size of 100nm in diameter (Figure 2A). Additionally, we further analyzed the diameter and size distribution of our exosome preparations using nanoparticle tracking analysis (NTA), which measures particles such as microvesicles (Figure 2E). Over 95% of all the particles diameter of particles was distributed from 50 to 120 nm in width, with the mean size as 93nm and 95nm in CSCs and DCs, respectively. Average diameter of DC-exosomes was relatively larger than their counterpart. Further, the centralized peak of NTA results indicated that the contamination of material derived from other cellular compartments in the exosomal fractions was minimal (Figure 2E). In order to confirm and validate isolated exosomes from our preparations, we investigated the presence of three typical known exosomal markers by western blot. The result in Figure 2B showed that the existence of CD63, CD81 and CD9 were clearly detectable by western blot with each corresponding band at 53kD, 70kD and 81kD, while these exosomal markers were absent in control. These observations and analyses verified the existence of exosomes in the preparation, and showed obvious characteristics of exosomes, with differences in size of exosomes compared between CSCs and DCs. Furthermore, we utilized Pkh-26 labeled exosomes to determine whether isolated exosomes still possessed biological activity between cells. Briefly, CSC-exosomes and DC-exosomes were separately incubated with Pkh-26 buffer, subsequently isolated for the second time as former transparent exosomes became visible red pellets. We administrated those labeled exosomes in the MKN45-GFP cells for 2.5 hours, followed by a thorough wash and fixation by 4% PFA. A confocal microscope was used to detect the signal of exosomes directly. As shown in Figure 2C, only CSC-exosomes were incorporated by MKN45 cells and were detectable in the cytoplasm, whereas DC-exosomes revealed no signal. More careful characterization was also performed to verify the internalization of CSC-exosomes into MKN45 cells. We treated MKN45 cells with 20-50 μg/ml of CSC-exosomes and, after 24h, analyzed the expression levels of miR-1290 in MKN45 cells as the exosomal abundance of miR-1290 was highest (Supplementary File 3). The level of miR-1290 was increased in a dose-dependent manner compared with untreated group. To exclude the possibility that CSC-exosomes could induce the endogenous microRNA expression, we further test the levels of precursor miR-1290 (pre-miR-1290) which showed no statistically significant difference between treatment and control group (Figure 2D). These results suggested that isolated exosomes still have vitality and could be an important communication material between cells, especially CSCs and their progeny. Next, we also interested in cell behavior changes after internalization of exosomes. After treatment with exosomes, MKN45 cell proliferation rate were calculated (CCK8) and stemness makers (CD44, CD133, Oct4, Sox2) were tested by western blot, but no significant changes were observed. The same results were also obtained in MKN74 cells (Supplementary Figure 2).

### Nucleotide composition of exosomes

**Figure 3:**
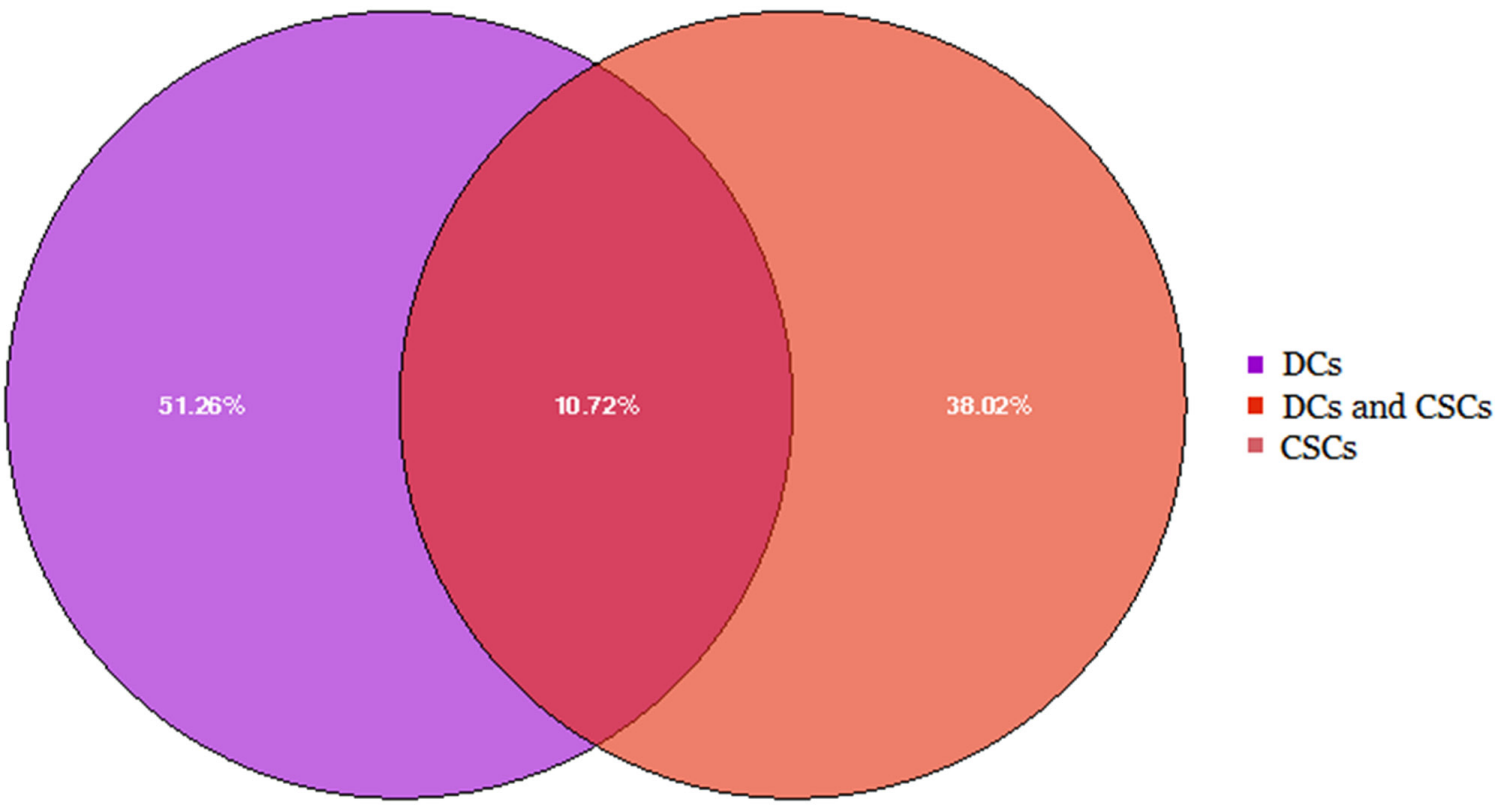
Comparison of total RNA reads in the exosomes from CSCs and DCs Result showed that common reads only account for 10.72%.

**Figure 4:**
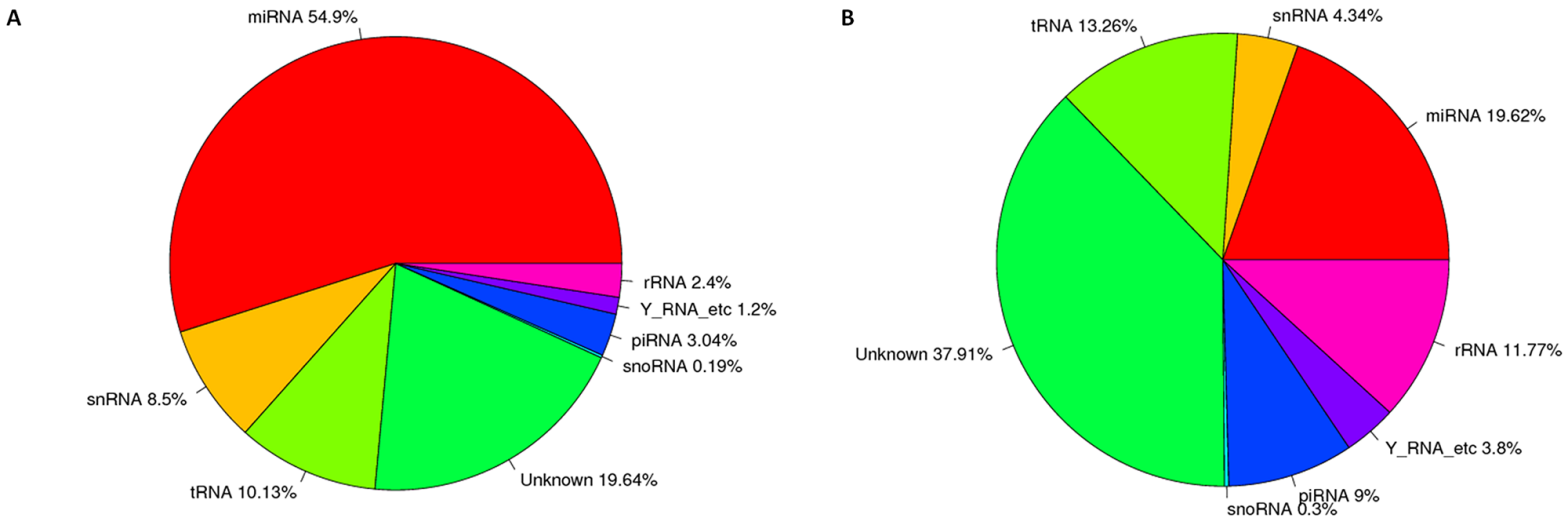
Nucleotide composition of exosomes from CSCs and DCs **(A)** The total RNA reads sequenced in the exosomes from CSC cells, showed the annotion of ncRNA. **(B)** The total RNA reads sequenced in the exosomes from DCs, showed the annotion of ncRNA. The database of miRBase (version:21), Rfam11.0 (rfam.janelia.org), UCSC (gtrnadb.ucsc.edu), pirnabank (http://pirnabank.ibab.ac.in/) were separately used for the annotion of miRNA, rRNA, snRNA, snoRNA, tRNA, piRNA. The percentage is calculated as a percentage of clean reads.

To characterize small RNAs in CSC- and DC-derived exosomes, Illumina HiSeq 2500 high-throughput technology was employed to sequence the two small RNA libraries. Initially, 17635958 and 16898706 raw reads were produced. According to Li, H. and R. Durbin [43], corresponding 15349221 and 10039436 clean reads were obtained after trimming low-quality reads and adaptor sequences. The results in Supplementary Table 1 and Figure 3 showed that total RNA reads were quite different in the CSC- and DC-derived exosomes. The unique RNA reads took about 38.02% and 51.26% of CSCs and DCs, while only 10.72% (203934) common RNA reads were found. We next mapped all clean reads to miRBase (v.21) to annotate known miRNAs in each library. The data in Supplementary File 1 and Supplementary Table 1 showed that 399 and 334 known miRNAs were identified in the exosomes from CSCs and DCs, respectively. In addition, 33 novel miRNAs and 37 novel pre-miRNAs were predicted in CSCs, whereas 123 novel miRNAs and 144 novel pre-miRNAs were predicted in DCs (Supplementary File 2). The clean reads identified for other small RNA categories (rRNA, tRNA, snRNA, snoRNA, srpRNA, repeat-associated RNAs, mRNA degradation) and unannotated RNAs are shown in Supplementary Table 1 and Figure 4. The percentage of miRNAs in the total RNA isolated from each sample corresponded to 54.90% and 19.62% for CSCs and DCs, respectively.

### Expression profiling of exosomal miRNAs

**Figure 5:**
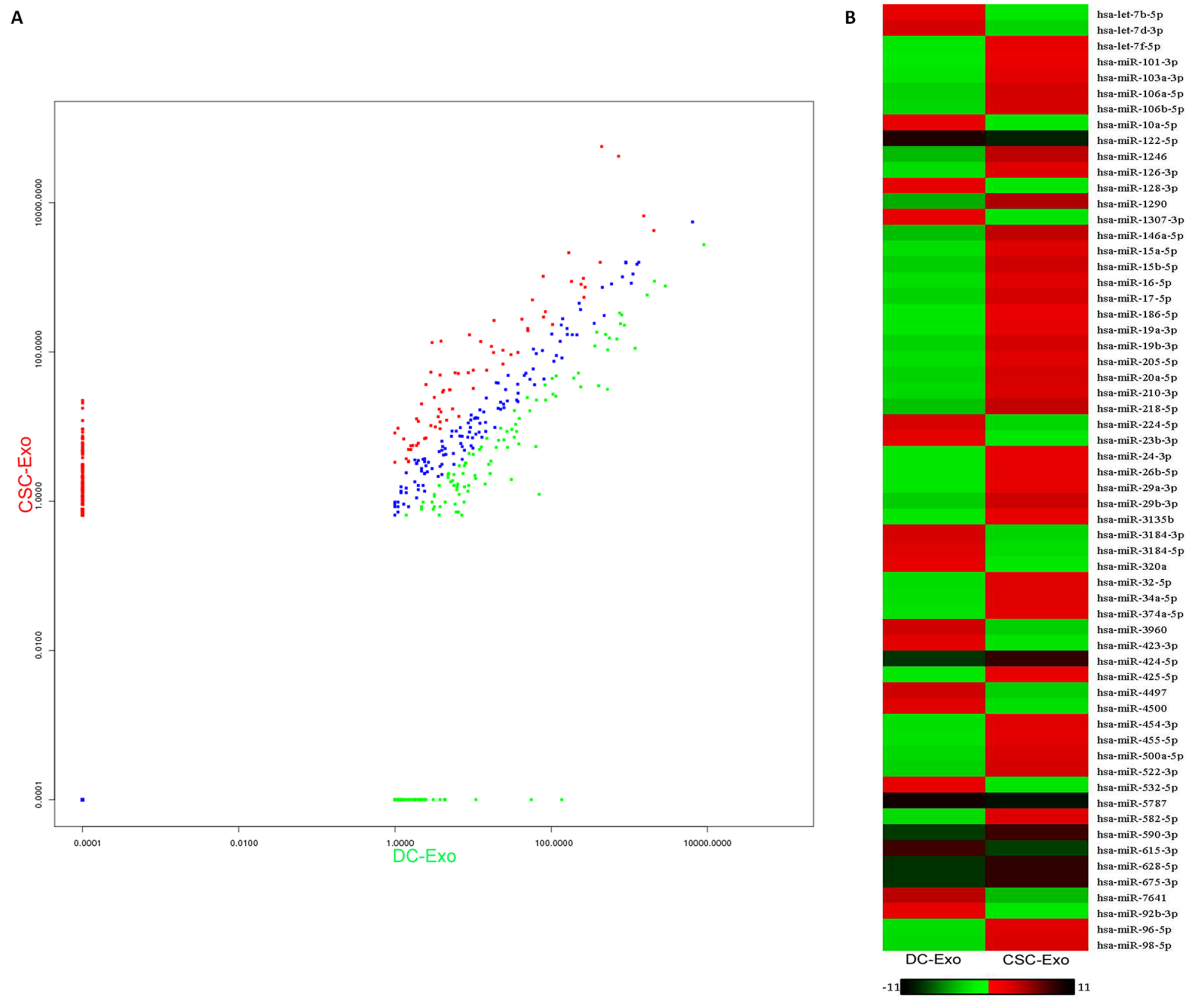
The differentially expressional pattern of exosomal miRNAs from CSCs and DCs **(A)** The diagram showed totally 309 differentially expressed miRNAs identified. Green, down-regulated miRNAs; blue, not differentially expressed miRNAs; red, up-regulated micRNAs. The criteria is a minimum 2-fold difference of log2 (fold change) in either direction. **(B)** The remarkable differentially expression of 60 miRNAs with a P-value<0.01, red, up-regulated micRNAs; green, down-regulated miRNAs.

**Figure 6:**
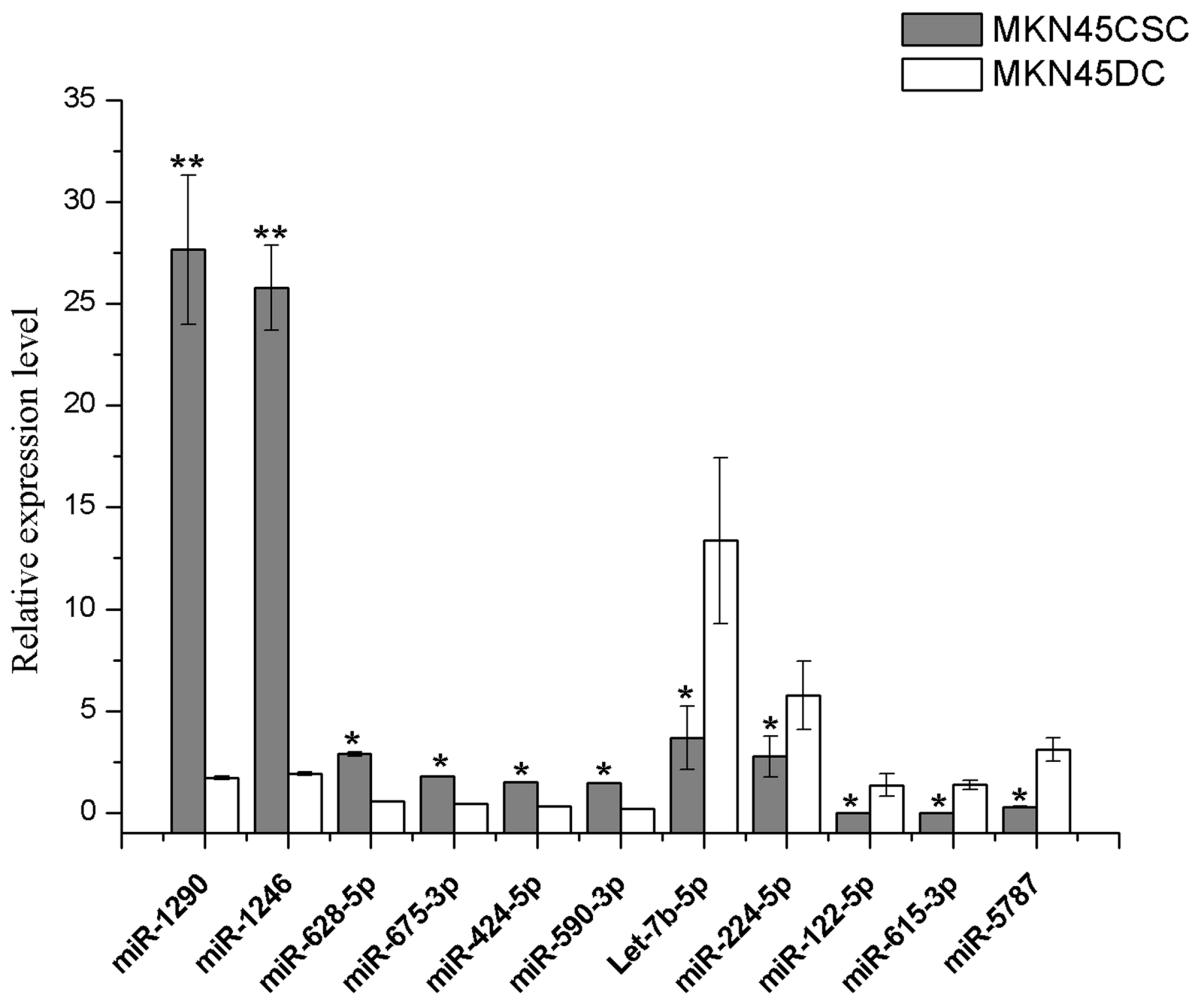
Expression of signature microRNAs Quantitative RT-PCR using Taqman miRNA assays was used to investigate the expression of 11 miRNAs in exosomes purified from CSC and DCs. The obtained values were normalized to hsa-miR-16a as an internal control. Grey, exosomal miRNAs from CSCs; white, exosomal miRNAs from DCs. Error bars show standard error of the mean (SEM). The experiments were repeated three times independently. ^*^P<0.05, ^**^P<0.01 compared with DCs.

We performed miRNA profiling to identify the differential miRNAs in the exosomes derived from CSCs, compared to DCs. The results of Deep sequencing of miRNA libraries showed that the highly expressed miRNAs were quite different among exosomes from CSCs and DCs (Supplementary File 3). In the exosomes of CSCs, 105 distinct miRNAs were found, and 15 miRNAs (including hsa-miR-1290, hsa-miR-1246, has-let-7f-5p, hsa-miR-21-5p, has-let-7a-5p, hsa-miR-100-5p, hsa-miR-20a-5p, hsa-let-7g-5p, hsa-miR-26a-5p, hsa-miR-24-3p, hsa-miR-182-5p, hsa-miR-378a-3p, hsa-miR-148a-3p, hsa-miR-17-5p, and hsa-miR-23a-5p) were significantly enriched, of which hsa-miR-1290 and hsa-miR-1246 were the most prominent with a 100-10000 fold up-regulated than the other identified miRNAs, while 11 different miRNAs (hsa-miR-100-5p, hsa-let-7b-5p, hsa-let-7i-5p, hsa-let-7a-5p, hsa-miR-92a-3p, hsa-let-7f-5p, hsa-miR-26a-5p, hsa-miR-378a-3p, hsa-miR-224-5p, hsa-miR-14a-3p, hsa-miR-191-5p) were highly expressed and hsa-miR-100-5p and hsa-let-7b-5p were the most prominent in the DC exosomes. We next compared the exosomal miRNA profiles to find the difference between CSCs and DCs. The results are shown in Figure 5A and Supplementary Figure 1. In total, 309 differentially expressed miRNAs were identified with a cut-off value of 2-fold difference according to the criteria in the methods section. Among them, 30 miRNAs showed differential levels of enrichment with 0.01<P-value<0.05, and 60 miRNAs with a P-value<0.01, in which 39 were up-regulated and 21 were down regulated in CSCs, compared to DCs (Figure 5B). Furthermore, according to the fold change ranking, typical differentially expressed miRNAs were identified as a miRNA signature, including 6 up-regulated miRNAs (miR-1290, miR-1246, miR-628-5p, miR-675-3p, miR-424-5p, miR-590-3p) and 5 down-regulated miRNAs (let-7b-5p, miR-224-5p, miR-122-5p, miR-615-3p, miR-5787). Among the up-regulated miRNAs, miR-1290 and miR-1246 were the most abundant in the exosomes from CSCs, while miR-628-5p, miR-675-3p, miR-424-5p, miR-590-3p were expressed distinctly in the exosomes from CSCs, compared to DCs. We next performed qRT-PCR to evaluated the expression levels of the signature miRNAs. As shown in Figure 6, miRNAs detected by qRT-PCR were consistent in expression with the deep sequencing results.

### Target gene prediction and GO and KEGG pathway enrichments of the predicted genes

For the purpose of investigating the influence of miRNAs in CSCs on gastric cancer, we predicted the target genes of the 11 signature miRNAs with the intersection of miRanda, miRDB and TargetScan and CLIP software. For mir-675-3p, there were no target genes predicted for TargetScan and CLIP, so we took the genes predicted by both miRanda and miRDB as target genes. Similarly, only miRDB was able to identifying the target genes for mir-5787, so the genes predicted by miRDB were considered as target genes. In total, 3363 target genes were found for the 11 signature miRNAs (Table 1).

**Table 1:** Number of target genes for selected miRNAs

Group	miRNA	Target genes	Target genes with GO
Up	hsa-miR-424-5p	118	110
	hsa-miR-590-3p	986	911
	hsa-miR-628-5p	233	161
	hsa-miR-675-3p	159	143
	hsa-miR-1246	192	159
	hsa-miR-1290	351	315
Down	hsa-let-7b-5p	11	8
	hsa-miR-224-5p	48	46
	hsa-miR-122-5p	87	76
	hsa-miR-615-3p	9	8
	hsa-miR-5787	1169	1070
Total		3363	3007

**Figure 7:**
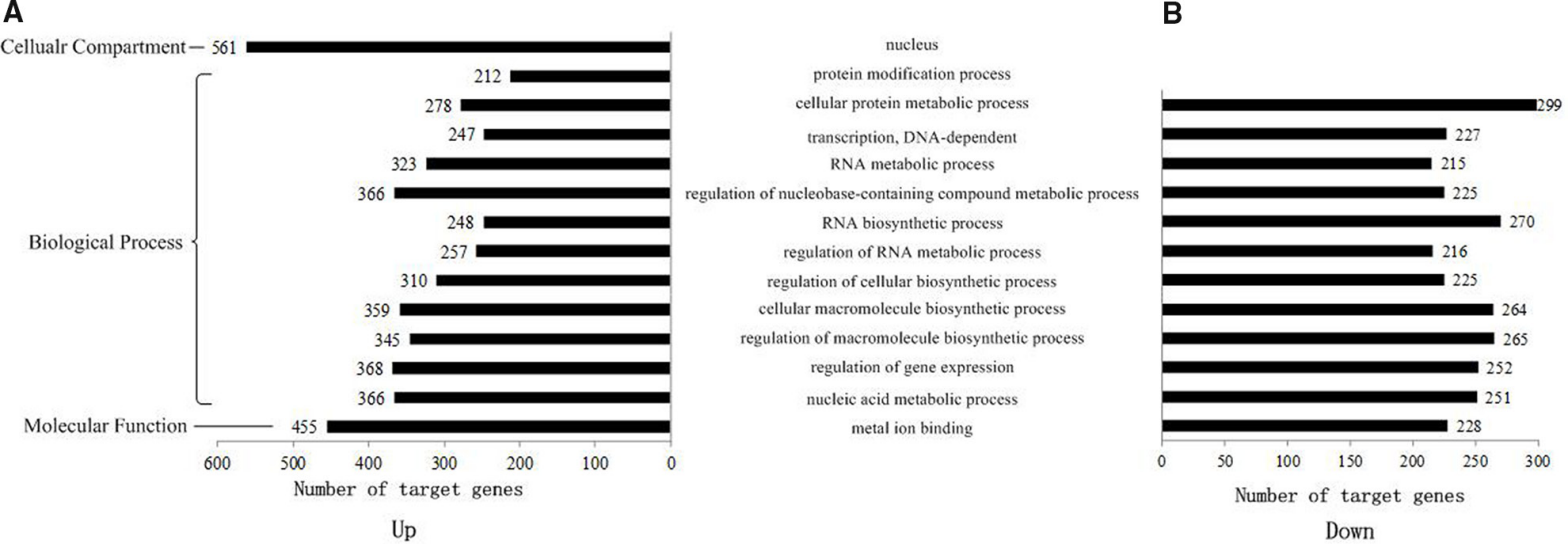
GO distribution of target genes in up- and down-regulated groups 11 selected signature miRNAs were classified by gene ontology in two groups. **(A)** Up-regulated group, **(B)** down-regulated group.

To comprehensively describe the properties of the targets, the putative genes were subjected to Gene Ontology (GO) enrichment and Kyoto Encyclopedia of Genes and Genomes (KEGG) pathway analysis. Not all the genes could be successfully annotated to GO items. Table 1 shows the number of target genes and those annotated to GO items in up-regulated and down-regulated groups. For these genes, we further investigated the GO and KEGG enrichment for two groups, respectively. The GO annotations with more than 20 target genes are shown in Figure 7. Target genes of CSCs and DCs were all enriched in nucleic acid metabolic processes, regulation of gene expression, regulation of macromolecule biosynthetic processes, cellular macromolecule biosynthetic processes, regulation of cellular biosynthetic processes, regulation of RNA metabolic processes, RNA biosynthetic processes, regulation of nucleobase-containing compound metabolic processes, RNA metabolic processes, and DNA-dependent transcription and cellular protein metabolic processes. Many genes were found to be metal ion binding genes. The most obvious result is that many target genes located in the nucleus were in the up-regulated group and participate in protein modification processes. Moreover, to find out if these signature miRNAs participate in any cancer hallmark process [44], we put together all eleven signature miRNAs. Then GO term enrichment analysis was conducted in all enriched target genes. As shown in Supplementary Figure 3, the result suggested these miRNAs are associated with two cancer hallmarks, regulation of cell death and apoptosis and regulation of cell proliferation.

**Figure 8:**
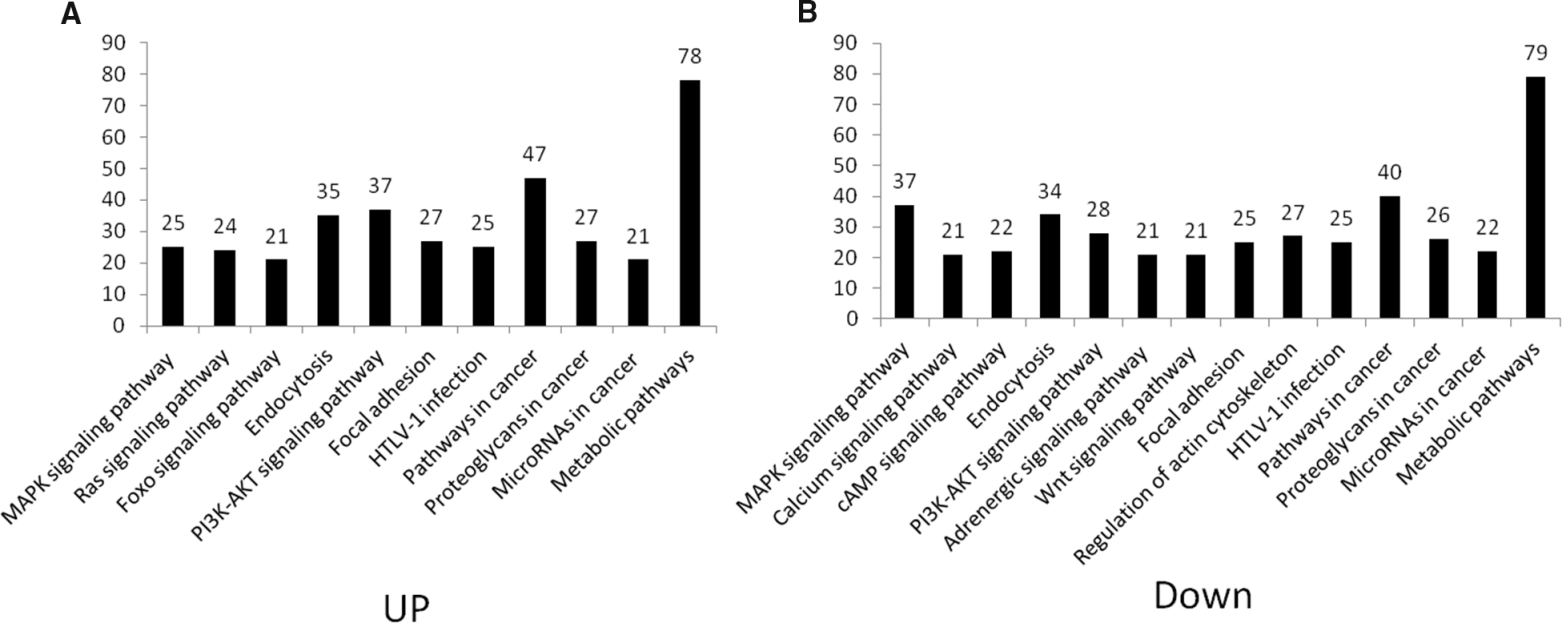
Pathway distribution of target genes in up- and down-regulated groups Predicted mRNA target of signature miRNA was subjected to KEGG pathway enrichment analysis. Pathways with more than 20 target genes in up- and down-regulated groups were selected, respectively. **(A)** Up-regulated group, **(B)** down-regulated group.

In the pathway analysis, pathways with more than 20 target genes in up- and down-regulated groups were selected (Figure 8). Target genes were enriched in the MAPK signaling pathway, endocytosis, PI3K-Akt signaling pathway, focal adhesion, HTLV-I infection, pathways in cancer, proteoglycans in cancer, microRNAs in cancer and metabolic pathways in both up- and down-regulated groups. In the up-regulated group, more target genes were found to participate in Ras and FoxO signaling pathways, but in the down-regulated group, more target genes were involved in calcium, cAMP, WNT signaling pathway, adrenergic signaling in cardiomyocytes and regulation of the actin cytoskeleton pathway.

### Validation of the exosomal signature miRNAs

**Figure 9:**
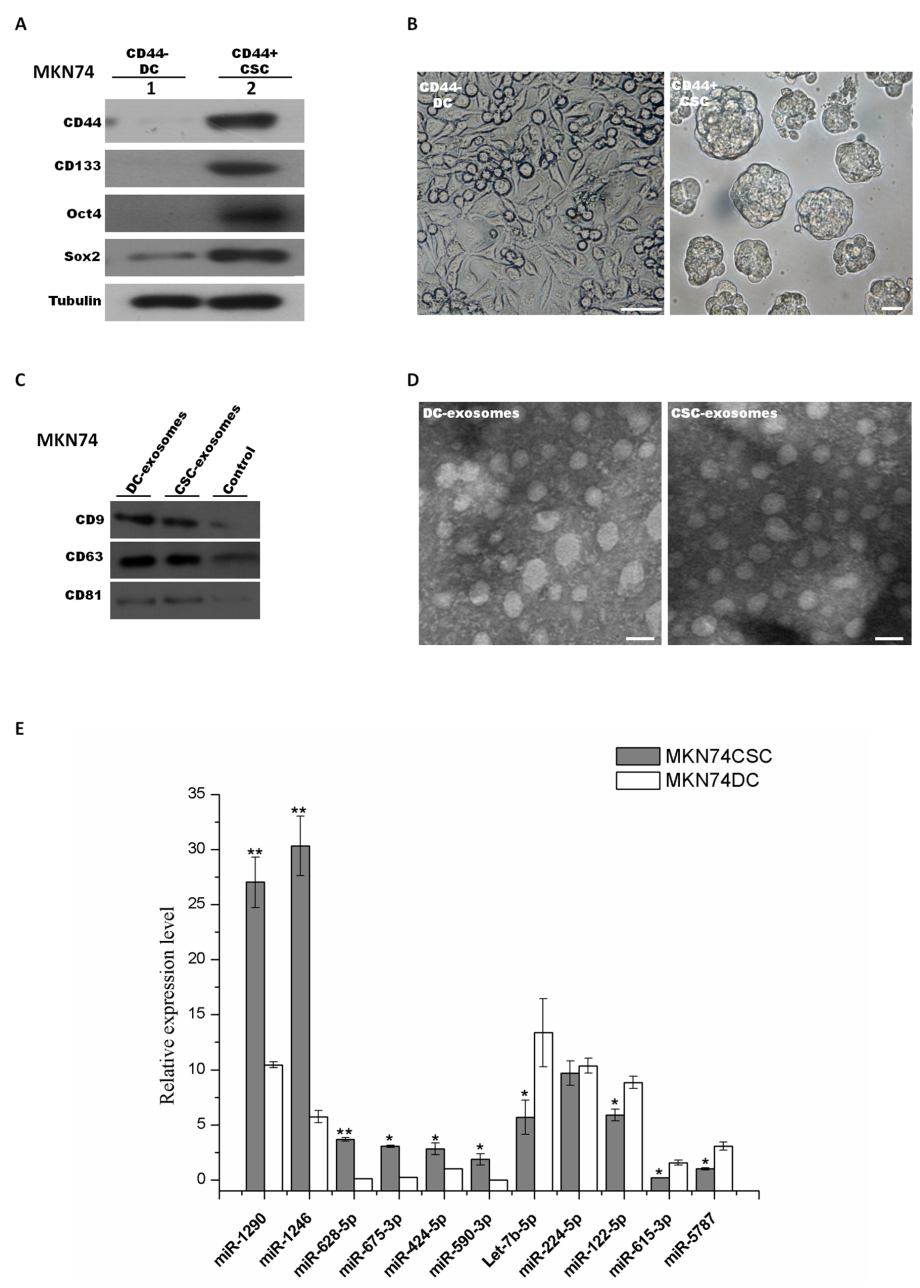
Exosomal signature miRNAs were validated in MKN74 derived CSCs and DCs MKN74 cancer stem-like cells were isolated and characterized. Signature miRNAs were further validated in MKN74 CSC-exosomes and DC-exosomes. **(A)** MKN74 derived CSCs and DCs were isolated and FACS-sorted from MKN74 cell line by using same strategy. Expression levels of CD44, CD133, Sox2 and Oct4 were determined by western blot between sorted MKN74 CSC population and DC population. **(B)** Formation of gastrospheres was observed in culture on day 8 (right) by plating CD44+ cells at clonal density. CD44- cells, represented for differentiated cells (DCs), were plated on adherent surface (left). Scale bars: 50 μm. **(C)** Western blot characterized exosomes derived from MKN74 CSCs and DCs using antibodies against exosomal protein marker (CD9, CD63 and CD81). **(D)** Electron micrograph of MKN74 CSCs and DCs exosomes. The scale bar indicates 100nm. **(E)** Quantitative RT-PCR was used to validate the expression of exosomal signature miRNAs in MKN74 CSC and DCs. The obtained values were normalized to hsa-miR-16a as an internal control. Grey, exosomal miRNAs from CSCs; white, exosomal miRNAs from DCs. Error bars show standard error of the mean (SEM). The experiments were repeated three times independently. ^*^P<0.05, ^**^P<0.01 compared with DCs.

To confirm the selected signature miRNAs, MKN74 were chosen to validate our finding. Initially, the same strategy was used to isolate and characterize MKN74-CSCs and MKN74-DCs (Figure 9A and 9B). Then MKN74 CSC-exosomes and DC-exosomes were isolated by use of ExoquickTM kit, and their purity was confirmed under a transmission electron microscope (Figure 9D). CD9, CD81 and CD63 were used to check the exosome isolation by western blot (Figure 9C). Then we used qRT-PCR assay to confirm the expression of 11 signature miRNAs which were selected from previous analyses. We identified ten miRNAs (i.e., miR-1290, miR-1246, miR-628-5p, miR-675-3p, miR-424-5p, miR-590-3p, miR-5787, let-7b-5p, miR-122-5p and miR-615-3p) showed the same differential expression. But we did not find significant difference in miR-224-5p (Figure 9).

**Figure 10:**
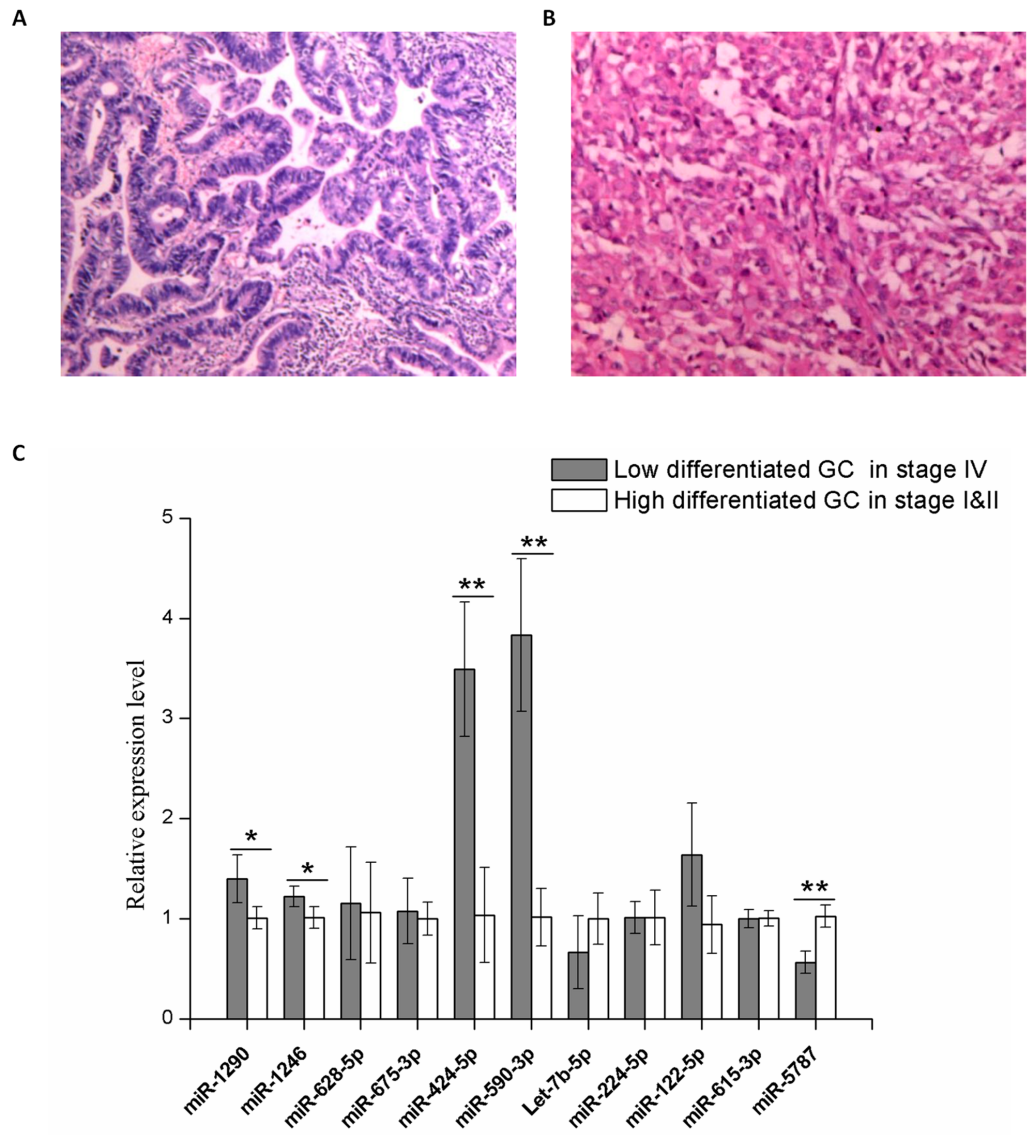
Validation of the serum levels of 11 signature miRNAs in exosomes A total of 12 patients were divided into two groups upon final pathology. **(A)** Representative biopsy result that was read as high-differentiated GC. **(B)** Representative biopsy result that was read as low-differentiated GC. **(C)** Quantitative RT-PCR using Taqman miRNA assay was used to investigate the expression of 11 signature exosomal miRNAs purified from GC patients serum. Grey, serum sample of stage IV GC with low differentiation (n=6); white, serum sample of stage I&II GC with high differentiation (n=6). Y axis was presented as relative expression (normalized with respect to miR-16a and compared with the average of reference sample in each group; 2^-ΔΔCt^). Error bars show standard error of the mean (SEM). The experiments were repeated three times independently. P-values adjusted for multiple testing by t-test were shown. ^*^P<0.05, ^**^P<0.01.

Along with the validation using MKN74 cells, serum samples from gastric cancer patients were collected to further confirm the effectiveness exosomal signature miRNAs. All samples were derived from Chinese GC patients who have signed the informed consent. From each patient, 10ml of venous blood was collected from each patient and serum was extracted within 1 hour, and then exosomes were isolated from each serum sample. The expression of exosomal signature miRNAs were then validated in a cohort of 12 GC samples, which comprised low-differentiated (Figure 9B) GC in stage IV (n=6) and high-differentiated GC (Figure 9A) in stage I&II (n=6). Five of the tested serum exosomal miRNAs exhibited differential expression, which were in agreement with results described above (Figure 10C).

Based on validation work in MKN74 cells and serum samples of GC patients, we propose five exosomal miRNAs (miR-1290, miR-1246, miR-424-5p, miR-590-3p and miR-5787) were validated miRNAs which retained relatively reliable prognostic significance. Of note, we only used 12 samples for the validation. Although the sample number is relatively small, 6 miRNAs have been validated. We expect that further validations will be done in future when we have more samples.

## DISCUSSION

A number of recent studies have demonstrated the presence of CSCs in gastric cancer, which share many characteristics with tissue stem cells, such as self-renewal and differentiation, and are responsible for sustaining the growth of tumors [7]. In this study, with the help of already established methods [12], we isolated CD44+ subpopulation cells in MKN45 as CSCs, which were subsequently identified by spheroid colony formation assay *in vitro*. The spherical colonies which grew for several weeks were considered indicative of self-renewal ability and consistent phenotype with CSC. This result proves the existence of stem-like cells in gastric cancer cell line MKN45, and is consistent with hierarchical organization of tumors.

For the accurate diagnosis of gastric cancer, researchers have made efforts to develop signature miRNAs in serum. However, the origin and function of these miRNAs have not been elaborated systematically. The ambiguous origin of the identified signature miRNA impedes the development and application of miRNAs as a non-invasive diagnostic marker for gastric cancer. Recently, it has been reported that miRNAs are mostly loaded and transported by exosomes, which are small membrane particles which may promote cancer aggressivity and metastatsis by transferring biological materials to other cells. The convenient isolation and stable features of exosomes make their components well conserved. Therefore, exosomal miRNAs have been commonly considered as an alternatively advanced signature for many diseases.

Our study showed that both CSCs and DCs secrete exosomes that exhibited abundant CD9, CD63 and CD81 expression, with miRNAs constituting the dominant components in exosomes of gastric CSCs. Furthermore, we determined that the exosomes from CSCs still possessed biological activity between cells. We also show for the first time that exosomes of gastric CSCs display a specific signature, characterized by differentially expressed exosomal miRNA clusters. In detail, 309 total distinct differentially expressed miRNAs were found between CSCs and DCs. In one study of gastric cancer, Sirjana Shrestha et al. [45] reviewed the miRNA profiles between gastric cancer and normal cells (NC), and finally collected 120 differentially expressed miRNAs which were reported in at least two studies. The CSC-exosome includes 7 of the 120 reported miRNAs and many more CSC-specific miRNAs, which shows the specificity of CSC in the GC studies. In CSCs, about 77% (309/399) miRNAs are distinctly differentially expressed with p<0.05 and fold change >2. This finding indicates much more difference between CSC and DC compared with GC vs. normal cells, which suggests that miRNA analysis is a useful approach for the identification of CSCs and DCs. As the appearance of CSCs indicates the signs of further development of GC advancing past the early stage, the remarkable difference between CSC vs DC and GC vs NC shows that the diagnosis of early occurrence and progression of GC are two different problems, which are likely characterized by different signatures.

Among the 309 distinct differentially expressed miRNAs between CSCs and DCs, 60 miRNAs showed differential levels of enrichment with a P-value<0.01 in which 39 were up-regulated and 21 were down-regulated in CSCs, compared to DCs. 11 signature miRNAs were selected and identified as most specific, including 6 up-regulated miRNAs (miR-1290, miR-1246, miR-628-5p, miR-675-3p, miR-424-5p, miR-590-3p) and 5 down-regulated miRNAs (let-7b-5p, miR-224-5p, miR-122-5p, miR-615-3p, miR-5787). Additionally, we also found that the highly expressed miRNAs were quite different in exosomes from CSCs and DCs, together with the obvious difference in the type and amount of miRNAs between CSCs and DCs. These remarkable distinctions identify the CSC exosomal miRNAs as an important potential signature for the certification of the stem-like features of the cells and the existence of CSCs. As CSCs indicate the development of GC, the signature set for identification of CSCs is potentially an effective biomarker for diagnosing the stage of GC progression.

With the further confirmation from Gene Expression Omnibus (GEO) dataset and literatures, we found that part of the 11 signature miRNAs have already been found or identified as biomarkers in GC and other kinds of cancers. MiR-1290 and miR-1246 has been proved to promote non-small cell lung cancer progression, and might be clinically useful as biomarkers for tracking disease progression and therapeutic targets [46]. miR-1290 has also been found as prognostic markers in castrate resistant prostate cancer [47]. In gastric cancer, miR-1290 was showed in positive correlation with clinical stages. miR-1290 was found to promote GC proliferation and metastasis through FOXA1 [48], which belongs to FoxO protein family that were manifested as an important enriched gene in this study (Figure 8). By targeting smad3, miR-424-5p was reported to be up-regulated in GC and promote GC proliferation *in vitro* and *in vivo* [49]. The miR-628-5p was significantly reduced in metastatic renal cell carcinoma patients compared to healthy controls [50]. MiR-5787 was found down-regulated in the exosomes released from camptothecin-treated hepatoma (data from GEO). These expression and clinical data shows the importance of these miRNAs to the diagnosis and progression of cancers, and thus the high possibility of their signature role in gastric cancer, which proves the reliability of our predictions.

To better understand how GC could be impacted by selected exosomal miRNA, 3363 total genes were predicted to be the targets of these biomarkers. Then, GO of the predicted target genes was annotated. Among the GO results, many genes were found to be metal ion binding genes. The most obvious result is that most of these target genes in the up-regulated group were located in the nucleus and participate in protein modification processes. Moreover, enrichment of regulation processes in both up- and down-regulated groups proved that the regulation abnormality might be an important cause of GC progression.

We then performed pathway enrichment analysis. The analyses indicated that the most significantly discrepant pathways associated with signatures are Ras and FoxO signaling pathways were in the up-regulated group, but calcium, cAMP, WNT signaling pathway, adrenergic signaling in cardiomyocytes and regulation of actin cytoskeleton were in the down-regulated group. These results suggest that CSC-exosomes contain distinct miRNA clusters which may reflect the accurate stage of GC progression. Upon transfering to neighboring cells, exosomes could promote tumor aggressiveness as well as shape their microenvironments. Indeed, Some of the enriched key pathways have been reported aberrantly regulated in GC and promoting GC progression, such as PI3K/AKT [51], MAPK/ERK [23, 52] and Ras signaling pathways [53]. Besides, FoxO family molecules, known be an important regulator of apoptosis and cell cycle in GC were investigated in studies [54, 55]. Focal adhesion kinase (FAK) and its gene amplification were found correlated with GC progression through stimulating GC cell migration and cancer invasion [56], and this process was found cross-talked to WNT signaling pathway [57]. Warburg effect on cancer metabolism is widely acknowledged. Recently, metabolome profiling was utilized to quantify metabolites changes in stomach tissue from GC patients and resulting in discovering several GC-specific anticancer targets [58]. However, little is known concerning cell-to-cell communication in GC. In this study, pathway analysis also provides implications on potential relationship between cell communication and underlying signaling pathways, such as cell recognition by proteoglycans, exogenous materials uptakes through endocytosis, signal transduction through second messager activation. Hence, these signaling pathways merit consideration as potential therapeutic targets in future research.

Next, validation work was conducted in MKN74 cells and GC patient serum samples. Due to the tumor heterogeneity (e.g., not the GC tumors have the same CSC origin) and potential differences between the CSC and it derived tumor cells (e.g., some of the key genes could be turned off during the transition between a CSC to matured tumor cells), we do not expect that all the signature miRNAs in the CSC study will be validated in the patient samples. As these exosomal signature miRNAs can differentiate high-grade GC from more progressive low-grade cancer in stage IV by testing obtainable serum (Figure 10), we suggest these signatures are non-invasive tools that can be potentially applied to cancer grading and/or cancer recurrence prediction.

In conclusion, data provided by this study might be invaluable for the identification of biomarkers to predict the progression of the disease to metastasis according to its CSC activity. Identification of signature miRNAs and further analyses of the functions of their target genes in this work offers novel alternative biomarkers for diagnosis and study of gastric cancer. The data are of great interest to the scientific gastric cancer community to further our understanding about the role of exosomes in gastric cancer progression.

## MATERIALS AND METHODS

### Patients and sample collection

All serum samples were obtained from GC patients at Second Affiliated Hospital of Tianjin University of TCM. Informed consent paper was obtained from all patients. The study was approved by the institutional review board, the Ethics and Indications Committee. Serum samples from 12 GC patients were collected. Clinicopathological factors and clinical stages were classified using the TNM system of classification. All data for the samples were obtained from the clinical and pathological records.

### Isolation and identification of gastric cancer stem-like cells (CSCs)

According to previously described methods [12], human gastric cancer cell line MKN45 was used to isolate gastric CSCs by flow cytometry analysis and fluorescence-activated cell sorting (FACS). MKN45 cells cultured in RPMI1640 medium (supplemented with 10% fetal bovine serum, 10 mM HEPES, and 1% of penicillin–streptomycin) were dissociated as single cells by trypsinization. For flow cytometry, cells were washed and incubated with the appropriate dilution FITC-conjugated anti-CD44 antibody (clone IM7; eBioscience company, San Diego, CA). After 45 minutes incubation at room temperature, cells were washed before analysis using either a FC500MCL or a BD FACSAriaII cytometer (Becton Dickinson). 1% CD44+ cells and CD44- cells were isolated and collected by cell sorting. CD44+ cells were then plated in serum-free medium supplemented with EGF, bFGF and at clonal density (1,000 cells/mL), and CD44- cells were plated in 30% exosome-depleted FBS with RPMI1640 medium containing an antibiotic–antimycotic. Subsequently, gastrosphere forming assays were used to characterize the CD44+ CSCs.

### Preparation of conditional medium (CM) and exosomes

We harvested the above medium used to culture CD44+ CSCs and CD44- DCs to isolate and purify exosomes. After incubation for 4 h, the CM was collected and centrifuged at 2,000 g for 10 min at 4 C. To thoroughly remove cellular debris, the supernatant was further centrifuged at 10,000g for 10min at 4 C. Total exosome isolation reagent (ExoQuick™ Exosome Precipitation Solution, System Biosciences) was utilized here to isolate and purify exosomes. The putative exosomes fraction was measured for its protein content using western blot.

### Transmission electron microscopy (TEM)

Purified exosome samples were diluted in a linear gradient and adsorbed onto formvar carbon-coated 300 mesh copper grids. After adsorption for 10 minutes, the samples were stained with 3% phosphotungstic acid for 1 minute, and then dried at room temperature for 20 minutes. Subsequently, the exosomes were observed under a transmission electron microscope (Jeol JEM-1230, JEOL Inc, Peabody, MA, USA) at 80 kV, and images of the exosomes were captured by a digital camera.

### Western blot analysis

The purified exosomal samples were lysed in SDS sample buffer for 20 minutes at room temperature, separated via SDS-PAGE and electrotransferred to PVDF membranes (Millipore Corp. Bedford, MA, USA). Membranes were blocked in TBS containing 5% nonfat milk at 28°C for 2 hours and then incubated separately with primary mouse anti-CD9, mouse anti-CD63, mouse anti-CD81 (at 1:1000, System Biosciences, USA) and Tubulin (1:1000, Beyotime, China) at 4°C overnight. After washing membranes using TBST (TBS containing 0.1% Tween-20), membranes were incubated with secondary antibody peroxidase labeled anti-mouse antibodies (1:1000, Beyotime, China) for 2 hours, then the membranes were washed with TBST 4 times. The bands were visualized by chemiluminescence using the ECL western blot analysis system (NOVEX ECL CHEMI SUBSTRATE, Life technologies).

### Nanoparticle-tracking analysis

The harvested exosomal pellets were resuspended in PBS and subjected to size and concentration measurement with NanoSight NS300 (Malvern Instruments, Westborough, MA), which is a type of nanoparticle tracking analysis (NTA) software that visualizes and analyzes particles in liquids by relating the rate of Brownian motion to particle size. The light scattered by the particles with laser illumination is captured by a digital camera, and the motion of each particle is tracked from frame to frame. The rate of particle movement is related to a sphere-equivalent hydrodynamic radius as calculated through the Stokes-Einstein equation. 5 30-second videos were used to measure the statistics of size and distribution with this software.

### Small RNA library construction and sequencing

The purified exosomes from CSCs and DCs were separately extracted for total RNA including the small RNA fraction using standard RNA extraction methodology. The quality and quantity of the isolated RNA was determined by the ND100 Nanodrop (Thermo Fisher), while RNA integrity was evaluated using the Agilent 2200 TapeStation (Agilent Technologies, USA) using an RIN^e^ above 7.0. Two small RNA libraries were constructed and sequenced with Illumina TruSeq deep sequencing technology. RNAs were ligated with 3’ RNA adapter, followed by a 5’ adapter ligation. Subsequently, the adapter-ligated RNAs were subjected to RT-PCR and amplified with a low cycle. Then the PCR products were size-selected by PAGE gel according to instructions of TruSeq® Small RNA Sample Prep Kit (Illumina, USA). The purified library products were evaluated using the Agilent 2200 TapeStation and diluted to 10 pM for cluster generation in situ on the HiSeq2500 single-end flow cell, followed by sequencing (1#x00D7;50 bp) on HiSeq 2500 platform. Image files generated by the sequencer were processed to produce digital quality data (raw FASTQ files).

### Bioinformatic analyses

The resulting raw data was filtered to generate clean reads (18–30 nt), and then annotated by aligning to miRBase 21 (http://www.mirbase.org), Rfam11.0 (http://www.rfam.janelia.org), UCSC (http://www.gtrnadb.ucsc.edu), pirnabank (http//pirnabank.ibab.ac.in/). The un-mapped RNA reads were used for prediction of novel miRNAs with Mireap software. Expression profiles of known miRNAs from CSCs and DCs were identified and compared. Significant miRNA changes were selected based on the following criteria: (i) statistical significance—miRNA expression changes were identified using a P-value threshold of 0.01; and (ii) fold change expression—a minimum 2-fold difference in either direction was required. The expression of miRNA was normalized as the number of reads per million (RPM) clean tags:

$$\mathrm{RPM}=\frac{\mathrm{number of reads mapping to miRNA}}{\mathrm{number of reads in clean data}}\;10^{6}$$

### Quantitative real-time PCR (validation)

Quantitative real-time PCR (qRT-PCR) was used to verify the expression changes of miRNAs. 0.5-1μg total microRNAs prepared from exosomes derived from CSCs and DCs were reverse transcribed using a TaqMan miRNA Reverse Transcription (RT) Kit from Applied Biosystems/Life Technologies with Megaplex RT Primers (Human Pool A and Pool B, Applied Biosystems). RT reaction conditions were thermally cycled under the following conditions: 30 minutes at 16°C, 30 minutes at 42°C, and 5 minutes at 85°C. The products were stored at −20°C for later use or immediately processed according to the manufacturer’s protocol. Quantitative PCR was performed in 96-well reaction plates with an ABI 7500 Fast Real-Time PCR System (Applied Biosystems, Foster City, USA). Each reaction was performed in a 20 μl volume system containing 1 μl of Taqman small RNA assay, 1.33 μl of product from reverse transcription, 10 μl of Taqman Universal PCR Master Mix (no AmpErase UNG) and 7.67 μl of nuclease-free water. Template-free controls were used to evaluate background signal. The qRT-PCR program consisted of incubation at 50°C for 2 minutes, 95°C for 10 minutes, followed by 40 cycles each of denaturation at 95°C for 15 seconds and annealing and extension for 60 seconds at 60°C. Each sample was run in three duplicates and relative quantification of miRNA expression was calculated using the 2-ΔΔCt method. The mean expression level of human endogenous control (RNU6B) was used as an internal control in all miRNA experiments to allow for the comparison of expression results. The relative miRNA expression levels were then calculated by the comparative threshold cycle (Ct) method (2−ΔCt).

### Target genes prediction of differentially expressed microRNAs

Four software tools (TargetScan, miRanda, CLIP, miRDB) were employed to predict target genes for selected miRNAs. Only targets that were found by three of the 4 tools were identified to be the target genes of miRNAs. Five computational prediction algorithms (TargetScan, miRanda, PITA, RNAhybrid and microTar) were used to predict targets of the significant changed miRNAs identified in the microarray analysis. Following a comparison of all datasets, a subset of genes that were targeted by more than four algorithms was generated.

### Gene ontology and KEGG pathway analysis

The putative genes were subjected to gene ontology (GO) enrichment and Kyoto Encyclopedia of Genes and Genomes (KEGG) pathway analysis with DAVID 6.7 software (http://david.abcc.ncifcrf. gov/home.jsp). Fisher’s exact test and χ test were used to select the significant GO categories and signaling pathways. The threshold of significance was defined by the P value, with P < 0.1 regarded as significant for GO and KEGG analysis, respectively.

## SUPPLEMENTARY MATERIALS FIGURES AND TABLES








