# Supplementary material for: MicroRNA expression profiling in exosomes derived from gastric cancer stem-like cells
Source: Oncotarget. 2017 Sep 27;8(55):93839–55. doi: 10.18632/oncotarget.21288 (PMC5706839; doi:10.18632/oncotarget.21288)
Supplement: Supplementary file 1 [file oncotarget-08-93839-s001.pdf]

MicroRNA expression profiling in exosomes derived from gastric cancer stem-like cells

SUPPLEMENTARY MATERIALS

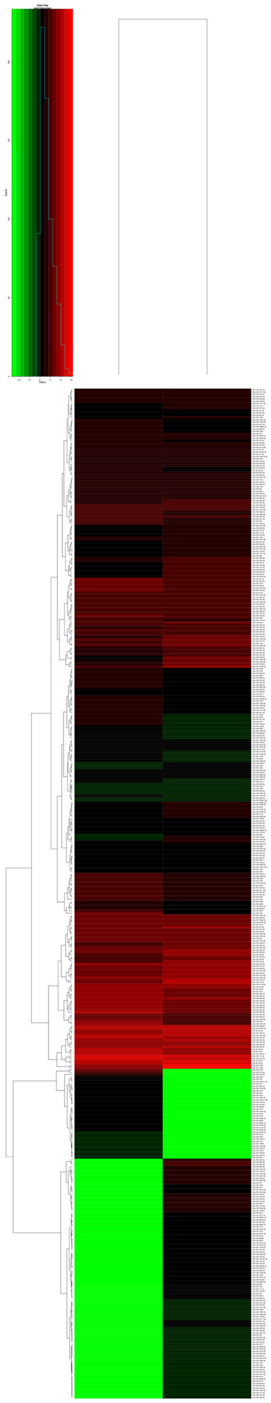

Supplementary Figure 1: Exosomal miRNAs profiling to find the difference between DCs (Left) and CSCs (Right).

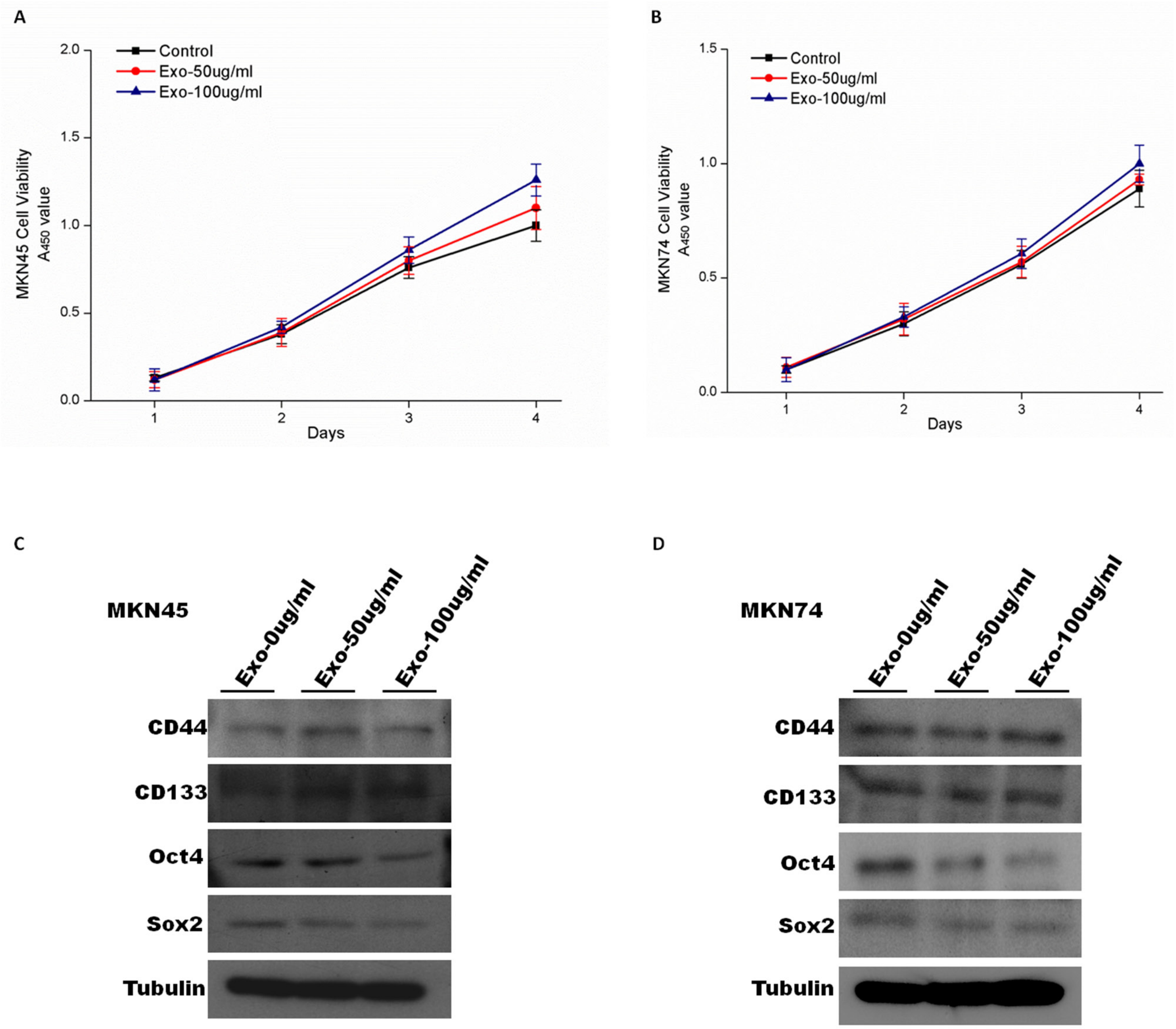

Supplementary Figure 2: MKN45 cell behavior changes by treatment of CSC-exosomes. A total of 5 × 10^3/well human gastric cancer MKN45 **(A)** and MKN74 **(B)** were seeded in 96-well microtiter plates. Cells were treated with CSC-exosomes at concentration of 0, 50, 100 ug/ml for 1-4 days. Then, cell proliferation was assessed by CCK8 assay. The cell proliferation was expressed as the mean ±S.D. for four independent wells of three independent experiments. **(C)** Stemness markers were tested in the MKN45 cells treated with different concentration of CSC-exosomes. **(D)** Stemness markers were tested in the MKN74 cells treated with different concentration of CSC-exosomes.

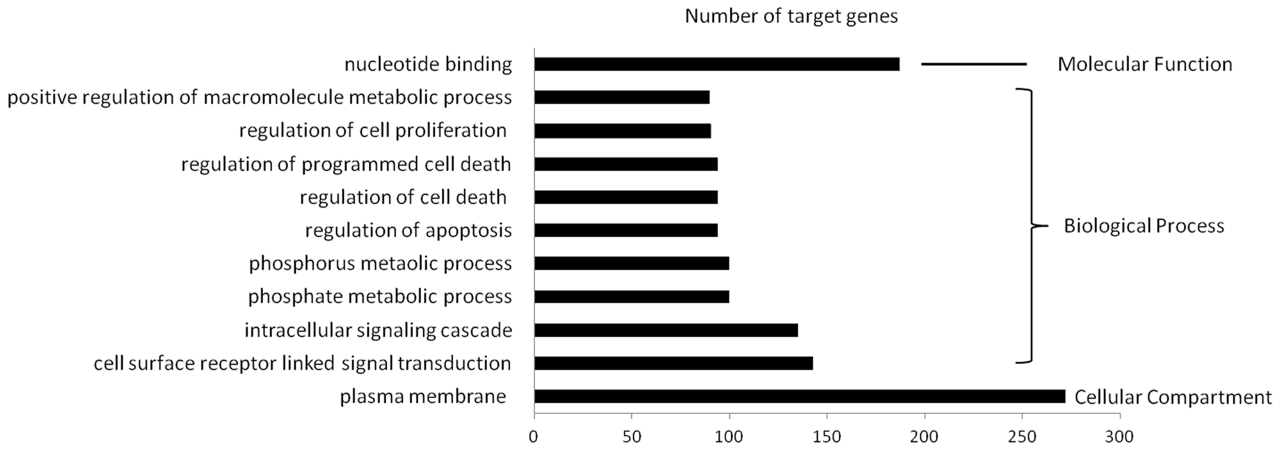

Supplementary Figure 3: GO term enrichment analysis for all signature miRNA target genes.

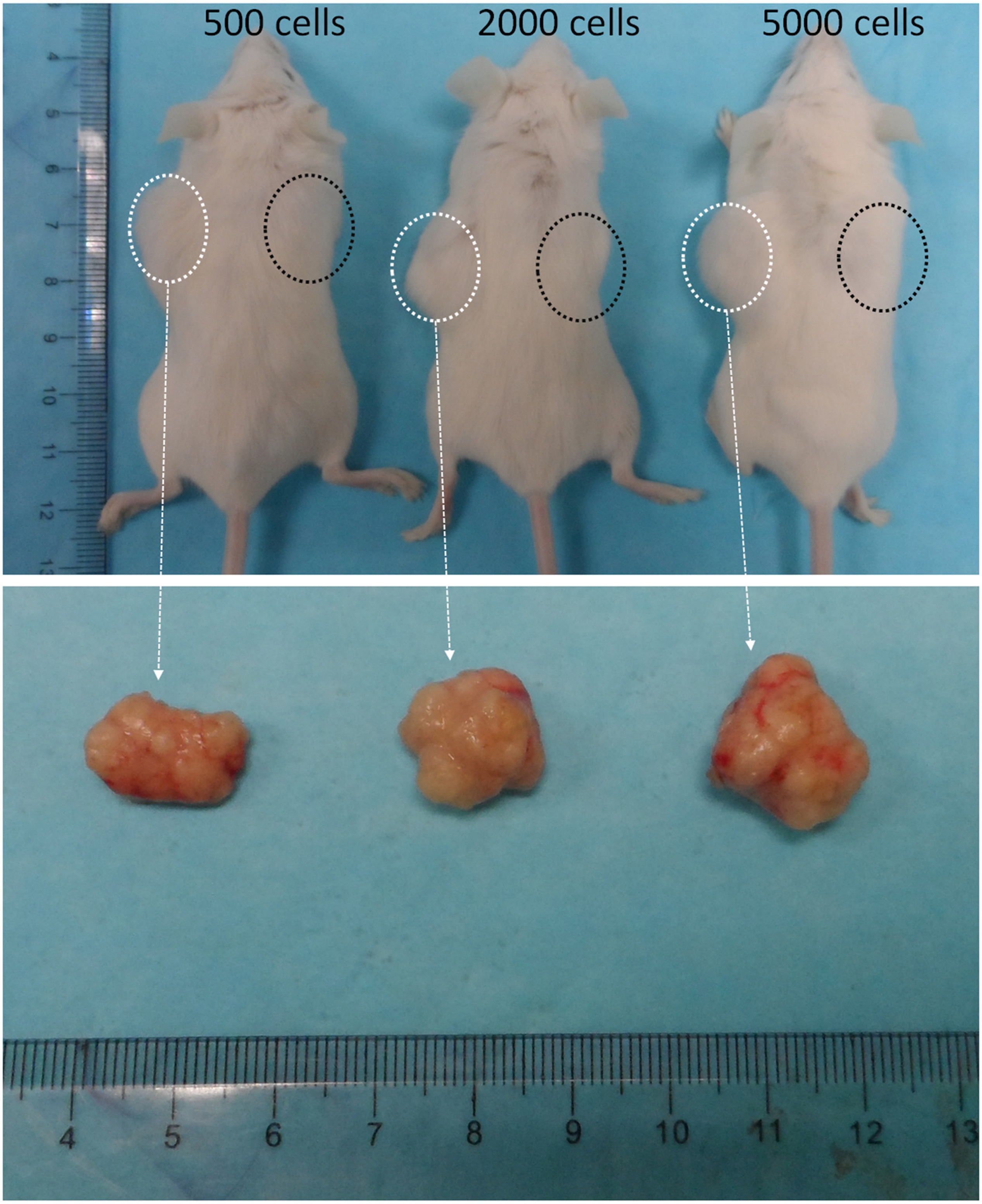

Supplementary Figure 4: Tumor formation of CSCs implanted in the skin of SCID mice. Sorted CD44 positive fraction (CSCs) implanted in the skin of SCID mice (500, 2000 and 5000 cells) produced tumors in each site after 8-12 weeks (white labels). While, sorted CD44- (DCs) fraction implanted in the skin of SCID mice produced no tumors (black labels).

Supplementary File 1: The known miRNAs in the exosomes derived from CSCs and DCs.

See Supplementary File 1

Supplementary File 2: The list of predicted miRNAs and pre-miRNAs.

See Supplementary File 2

Supplementary Table 1: Summary of small RNA sequencing of exosomes from DCs and CSCs

	DCs		CSCs	
Raw Reads	16898706		17635958	
GC%	53.42		50.62	
Clean reads[a]	10039436	59.41%	15349221	87.03%
Mapping to genome[b]	9339723	93.03%	14692871	95.72%
Mapping to mirBase[b]	1969827	19.62%	8426263	54.90%
rRNA etc. (rRNA, tRNA, snRNA, snoRNA, srpRNA)[b]	4264011	42.47%	5410345	35.25%
Known miRNA[b]	334		399	
New miRNA prediction[b]	123		33	
New pre-miRNA prediction	144		37	

[a]calculated as a percentage of raw reads.
[b]calculated as a percentage of clean reads

Supplementary File 3: The differentially expressed miRNAs in the exosomes derived from CSCs and DCs.

See Supplementary File 3
